# A New Long-Lasting Indoor Residual Formulation of the Organophosphate Insecticide Pirimiphos Methyl for Prolonged Control of Pyrethroid-Resistant Mosquitoes: An Experimental Hut Trial in Benin

**DOI:** 10.1371/journal.pone.0069516

**Published:** 2013-07-23

**Authors:** Mark Rowland, Pelagie Boko, Abibatou Odjo, Alex Asidi, Martin Akogbeto, Raphael N’Guessan

**Affiliations:** 1 London School of Hygiene & Tropical Medicine, London, United Kingdom; 2 Centre de Recherche Entomologique de Cotonou, Laboratoire Nationale, Ministère de la Santé, Cotonou, Benin; University of Crete, Greece

## Abstract

**Background:**

Indoor residual spraying (IRS) is widely used for malaria transmission control in sub-Saharan Africa. Resistance to pyrethroids in the mosquito *Anopheles gambiae* is a growing problem. There is an urgent need to develop long-lasting alternative insecticides to reduce selection pressure for pyrethroid resistance and to provide control with a single IRS application in countries with long transmission seasons.

**Methods:**

Two capsule suspension formulations (CS) of the organophosphate pirimiphos methyl were evaluated as IRS treatments in experimental huts in an area of Benin where the mosquitoes *Anopheles gambiae* and *Culex quinquefasciatus* are resistant to pyrethroids but susceptible to organophosphates. The CS formulations were tested alongside an emulsifiable concentrate (EC) formulation of pirimiphos methyl and a CS formulation of the pyrethroid lambdacyhalothrin.

**Results:**

The two CS formulations of pirimiphos methyl gave prolonged control of *An. gambiae* and *Cx. quinquefasciatus*. In cement huts application rates of 0.5 g/m^2^ induced high mortality of *An. gambiae* for almost a year: overall mortality rates 87% (95% CI 82–91%) and 92% (95% CI 88–94%). In mud huts application rates of 1 g/m^2^ induced high mortality of *An. gambiae* for 10 months: overall mortality rates 75% (95% CI 69–81%) and 76% (95% CI 68–83%). The EC formulation of pirimiphos methyl failed to control *An. gambiae* two months after spraying. The pyrethroid lambdacyhalothrin demonstrated prolonged residual activity in bioassay tests but failed to control pyrethroid resistant *An. gambiae* that entered the huts. Pirimiphos methyl CS was highly active against *Culex quinquefasciatus* and gave control for 10 months in cement huts and 6 months in mud huts.

**Conclusion:**

Pirimiphos methyl CS (Actellic 300 CS) applied at 1 g/m^2^ shows great promise for providing prolonged control of pyrethroid-resistant *An gambiae* and for delaying pyrethroid resistance. An alternative to DDT, giving year-round transmission control in sub-Saharan Africa is now a realistic prospect.

## Background

Malaria control based on long-lasting insecticidal nets (LLINs) and indoor residual spraying (IRS) is being scaled up across sub-Saharan Africa with support from the Global Fund (GFATM), the President’s Malaria Initiative (PMI), the UK Department for International Development and national governments [1]. In areas of seasonal transmission, prone to epidemics, IRS has the advantage of protecting entire populations at risk [2], [3]. In holendemic areas of more stable transmission two or more spray rounds of IRS may be required to cover the full transmission season [4], [5]. Spiralling costs of recurrent campaigns may make IRS with conventional formulations harder to justify or sustain [6]. Of the residual insecticides approved by WHO the longest lasting is DDT [2]. While the organophosphate, carbamate and pyrethroid classes of insecticide are more acceptable environmentally they are more expensive per unit area sprayed and shorter lived than DDT [2], [7]. Development of safer, more residual insecticides is paramount to future malaria control. Reformulation of WHO approved insecticides from the existing portfolio of insecticides offers the most pragmatic solution [8]. Microencapsulation of pyrethroids and organophosphate insecticides is both feasible and economic and can extend the residual life of insecticides when applied to cement or mud plastered rooms [9]–[11]. With the alarming increase in pyrethroid resistance reported from many endemic African countries [12]–[17], alternative insecticides from non-pyrethroid classes require urgent development, evaluation, approval and registration.

Pirimiphos-methyl is an organophosphate which in early field trials of an emulsifiable concentrate formulation (EC) demonstrated high level but short lived activity against anophelines and culicines [18]–[20]. The manufacturer of pirimiphos methyl, Syngenta, has recently developed microencapsulated formulations (CS) in response to the need to control pyrethroid resistant strains with a long lasting effect.

We report on an evaluation of pirimiphos methyl for indoor residual treatment conducted in experimental huts in southern Benin. The efficacy of two capsule suspension formulations of pirimiphos methyl (Actellic CS, Basle, Switzerland) was compared to a standard emulsifiable concentrate formulation (Actellic 50EC, Basle, Switzerland) and to the pyrethroid lambdacyhalothrin (Icon 10CS, Basle, Switzerland) in an area where *Anopheles gambiae* and *Culex quinquefasciatus* are difficult to control with pyrethroids or DDT owing to resistance [21], [22].

## Materials and Method

### Study Sites and Experimental Huts

The Akron field site is situated in a horticultural area on the outskirts of Porto Novo, the administrative capital of Benin. The site provides permanent breeding of pyrethroid resistant *Anopheles gambiae* M form, which contains *kdr* and metabolic resistance mechanisms [23], 2008). *Culex quinquefasciatus* is also abundant and is resistant to pyrethroids and DDT [24]. The experimental huts are made of concrete bricks, with roofs of corrugated iron, ceilings lined with woven palm matting on the interior surface, and walls coated with cement/sand or mud/sand plaster mixes [25]. Each hut stands on a concrete base surrounded by a narrow moat to exclude scavenging ants. Entry of mosquitoes occurs via four window slits, 1 cm wide, located on three sides of the hut. Mosquitoes exit into a verandah trap projecting from the fourth side.

### Insecticide Treatments

The insecticides produced by Syngenta were:

- pirimiphos methyl B 30% CS
- pirimiphos methyl BM 30% CS
- pirimiphos methyl 50% EC
- lambdacyhalothrin 10% CS

The following treatments and target application rates were compared in 12 experimental huts, some with cement plastered walls, others with dried mud plastered walls, at the Akron site:

- pirimiphos methyl B CS, 0.5 g/m^2^ (one mud and one cement walled hut)
- pirimiphos methyl BM CS, 0.5 g/m^2^ (one mud and one cement walled hut)
- pirimiphos methyl B CS, 1 g/m^2^ (one mud and one cement walled hut)
- pirimiphos methyl BM CS, 1 g/m^2^ (one mud and one cement walled hut)
- pirimiphos methyl EC, 1 g/m^2^ (one mud and one cement walled hut)
- lambdacyhalothrin CS, 0.03 mg/m^2^ (one cement walled hut)
- unsprayed control (one cement walled hut)

Hut interior surfaces were sprayed with an aqueous solution of insecticide applied using a Hudson XPert compression sprayer to the walls and the palm thatch ceilings. Spraying was completed on 16 April 2009. The trial started 3 days later and ran for 12 months.

### Trial Procedure

The trial followed WHO Pesticide Evaluation Scheme procedures for IRS [25]. Adult volunteers of 18 years or older were selected as volunteers by lot from the local village to sleep in the huts overnight. Volunteers were provided with chemoprophylaxis and the risks of malaria were explained. Written informed consent was obtained from all volunteer sleepers and documented. Volunteers were given basic remuneration for participating in the study. It was explained they had the right to withdraw to withdraw from the trial at any time without penalty.

Each treatment was dedicated to a particular hut. Volunteer sleepers rotated at random between huts, spending one night in each hut during the weekly rotation. Sleepers were interviewed individually using a questionnaire for possible adverse effects due to insecticide; this was done daily during the first week, weekly during the first month, and each month during the study year. Responses were documented for further analysis. Any volunteer showing adverse effect or sign of fever was referred to a local physician and, if malaria was confirmed, treated with Co-artem (artemether/lumefantrine, Novartis Pharmaceuticals corporation, USA).

Each morning mosquitoes were collected from the verandahs and rooms of huts and recorded as fed or unfed and dead or alive. Live mosquitoes were provided with 10% glucose solution and held for 24 h before scoring final mortality.

The trial protocol and consent form obtained approval from the ethical committees of LSHTM (application approval number 5256) and Ministry of Health, Cotonou, Benin MOH ethics committees.

The efficacy of each treatment was expressed in terms of:

Exiting rates: proportion of mosquitoes exiting and trapped in the verandah of a treated hut relative to the proportion in the control hut.
Blood-feeding inhibition: proportion of mosquitoes that were blood fed in sprayed huts relative to the proportion in control huts.
Mortality: proportion of mosquitoes found dead after 24 h holding time.

### Residual Activity

Each month of the trial the residual activity of each treatment on walls and ceilings was monitored by means of bioassay using WHO cone tests. The insecticide-susceptible *An. gambiae* Kisumu was used for this purpose, with exposure of 30 min and 24 h recovery as per WHO guideline [25].

### Species and Resistance Characterisation

Samples of An. gambiae s.l. reared from larval collections near the trial site were identified to species using PCR [26] and to molecular form using PCR RFLP [27].

WHO test kits lined with test papers treated with pirimiphos-methyl and deltamethrin in silicon oil were used to determine susceptibility of *An. gambiae* and *Cx. quinquefasciatus* females reared from larval collections [28]. Batches of unfed females, aged 2–5 days, were exposed to test papers for 1 h and mortality rates were recorded after 24 h. A discriminating concentration of pirimiphos-methyl which would kill 100% of susceptible insects was established using a 0.125–1.0% concentration range.

PRC diagnostic test for detection of *kdr* mutations was carried out on *An. gambiae* and *Cx. quinquefasciatus* mosquitoes as described by Martinez-Torres et al. [29]. The PCR-RFLP diagnostic test was used to detect the presence of the *Ace.1R* G119S mutation as described by Weill et al. [30].

### Data Analysis

The experimental hut data were entered in Excel and transferred to STATA 6.0 software for analysis. The outcomes of interest were the proportions of each species dying, blood-feeding, and exiting a treatment over time. Logistic regression for grouped data was used to estimate the outcomes between treatments, comparing results for formulation, dosage, substrate treated and period after treatment, with adjustment for clustering by day and for variation between individual sleepers. The decay of treatments over time in residual bioassay tests were analysed using grouped logistic regression comparing percentage mortality at intervals after spraying.

## Results

### Efficacy of IRS Against *An. gambiae* and *Cx. quinquefasciatus* after 12 Months

#### Huts with cement walls

Table 1 shows the entomological outcomes for *An. gambiae* and *Cx. quinquefasciatus* in huts with cement lined walls. Owing to a potential hut position effect (identified by the differences in number of mosquitoes collected from individual huts during preliminary mosquito collections) and the inability to rotate IRS treatments between huts, it was not possible to interpret numbers of mosquitoes entering the huts in terms of treatment effects. Overall there were fewer mosquitoes entering the pirimiphos methyl huts than untreated control or lambdacyalothrin huts but no inference can be drawn because treatment induced deterrence cannot be distinguished from differential attractiveness due to site position.

**Table 1:** Summary results of indoor residual spray (IRS) treatments against *An.gambiae* and *Cx. quinquefasciatus* over 12 months in cement lined experimental huts.

Cement huts	Total collected	% Caught in Veranda (CI)	% Bloodfed (CI)	% Mortality (CI)	% CorrectedMortality(CI)
***Anopheles gambiae***					
Untreated	1286	42^a^ (38–46)	89^a^ (85–92)	10^a^ (8–13)	–
P-methyl CS B 0.5 g/m^2^	914	40^a^ (36–44)	93^b^ (91–95)	88^b^ (82–91)	86 (80–89)
P-methyl CS BM 0.5 g/m^2^	964	40^a^ (36–44)	95^b^ (93–97)	92^bc^ (88–94)	91 (87–93)
P-methyl CS B 1 g/m^2^	855	39^a^ (35–43)	91^ab^ (86–94)	90^b^ (83–95)	89 (82–94)
P-methyl CS BM 1 g/m^2^	758	39^a^ (35–44)	91^ab^ (88–94)	95^c^ (93–97)	94 (92–96)
P-methyl EC 1 g/m^2^	841	37^a^ (32–43)	93^b^ (89–96)	26^d^ (21–32)	18 (13–24)
Lambdacyhalothrin CS	1149	64^b^ (61–69)	93^b^ (90–96)	22^d^ (18–28)	13 (9–19)
***Culex quinquefasciatus***					
Untreated	6178	39^a^ (37–42)	69^a^ (65–72)	9^a^ (8–11)	–
P-methyl CS B 0.5 g/m^2^	4661	35^a^ (33–37)	78^b^ (74–80)	56^b^ (49–61)	51 (46–49)
P-methyl CS BM 0.5 g/m^2^	4544	38^a^ (35–40)	74^b^ (71–77)	66^c^ (61–72)	64 (59–70)
P-methyl CS B 1 g/m^2^	4134	38^a^ (35–40)	71^a^ (67–75)	63^c^ (56–69)	60 (53–66)
P-methyl CS BM 1 g/m^2^	4059	36^a^ (34–39)	77^b^ (73–80)	73^d^ (68–77)	71 (66–75)
P-methyl EC 1 g/m^2^	3226	37^a^ (34–40)	76^b^ (72–79)	18^e^ (15–21)	10 (7–9)
Lambdacyhalothrin CS	4699	40^a^ (39–43)	73^a^ (69–76)	13^a^ (11–16)	5 (3–8)

Results in columns not sharing the same superscript are significantly different at the 5% level.

The proportions of *An. gambiae* and *Cx. quinquefasciatus* exiting into the verandahs of IRS treated huts by dawn were not significantly different to the proportions exiting the untreated control huts; the only exception was the higher proportion of *An. gambiae* exiting the lambdacyhalothrin-treated hut (p = 0.02) (table 1).

Blood-feeding rates of *An. gambiae* ranged from 91 to 95% and those of *Cx. quinquefasciatus* ranged from 71 to 78% across treatments. Because mosquitoes tend not to alight on treated walls or ceilings until after feeding, the IRS treatments tended not to affect blood feeding rates which, consequently did not differ between treatments.

In the cement walled huts the percentage mortality of *An. gambiae* was 88% for the 0.5 g/m^2^ and 90% for the 1.0 g/m^2^ applications of pirimiphos methyl CS B. In the pirimiphos methyl CS BM sprayed hut, mortality was 92% for the 0.5 g/m^2^ and 95% for 1.0 g/m^2^ applications. The differences in mortality between low and high application rates were not significant for either the pirimiphos methyl B or BM variants but overall the BM treatments killed significantly higher proportions of *An. gambiae* than did the B treatments (p<0.05) (table 1). Mortality rates of *An. gambiae* exposed to lambdacyhalothrin CS (22%) and pirimiphos methyl EC (26%) treatments were much lower than with the pirimiphos methyl CS treatments (p = 0.01).

The mortality of *Cx. quinquefasciatus* was consistently lower than *An. gambiae* mortality across all treatments. Nevertheless, over 12 months of the trial, more than 50% of the *Cx. quinquefasciatus* entering the huts were killed by each of the pirimiphos methyl CS treatments. The formulation that killed more *Cx. quinquefasciatus* (73%) than any other treatment was the 1.0 g/m^2^ application rate of CS BM (p<0.01) (table 1). Pirimiphos methyl EC and lambdacyhalothrin CS killed no more than 18% overall.

#### Huts with mud walls

Table 2 records the summary results for *An. gambiae* and *Cx. quinquefasciatus* over 12 months in huts lined with dried mud. The proportions of *An. gambiae* and *Cx. quinquefasciatus* exiting the treated huts were not significantly different from the proportions exiting the control hut. The proportions blood-feeding in the insecticide treated huts were not significantly different from proportions blood-feeding in the untreated control (table 2).

**Table 2:** Summary results of indoor residual spray (IRS) treatments against *An.gambiae* and *Cx. quinquefasciatus* over 12 months in mud lined experimental huts.

Mud hut	Total collected	% Caught in verandah (CI)	% Bloodfed (CI)	% Mortality (CI)	% Correctedmortality(CI)
***Anopheles gambiae***					
Control	1270	42^a^ (38–46)	89^a^ (85–92)	10^a^ (8–13)	–
P-methyl CS B 0.5 g/m^2^	976	38^a^ (34–42)	94^b^ (92–96)	42^b^ (37–49)	36 (31–43)
P-methyl CS BM 0.5 g/m^2^	882	37^a^ (34–42)	95^b^ (93–97)	54^c^ (46–61)	48 (40–55)
P-methyl CS B 1 g/m^2^	670	38^a^ (34–41)	97^b^ (95–98)	75^d^ (69–81)	72 (66–78)
P-methyl CS BM 1 g/m^2^	926	38^a^ (33–43)	97^b^ (95–98)	76^d^ (68–83)	73 (65–80)
P-methyl EC 1 g/m^2^	663	39^a^ (35–43)	95 (93–97)	23^e^ (18–28)	14 (9–19)
***Culex quinquefasciatus***					
Control	6145	39^a^ (37–42)	69^a^ (65–72)	9^a^ (8–11)	–
P-methyl CS B 0.5 g/m^2^	5178	37^ab^ (35–40)	80^b^ (76–83)	24^b^ (21–27)	16 (13–19)
P-methyl CS BM 0.5 g/m^2^	4909	34^b^ (32–36)	84^b^ (82–86)	29^c^ (26–33)	22 (19–26)
P-methyl CS B 1 g/m^2^	4797	33^b^ (31–35)	85^b^ (83–87)	46^d^ (42–50)	40 (36–44)
P-methyl CS BM 1 g/m^2^	5419	34^b^ (32–37)	83^b^ (80–85)	51^e^ (47–56)	46 (42–51)
P-methyl EC 1 g/m^2^	6046	36^a^ (34–39)	81^b^ (78–83)	15^f^ (13–17)	7 (5–9)

Results in columns not sharing the same superscript are significantly different at the 5% level.

The proportions of *An. gambiae* and *Cx. quinquefasciatus* killed in the mud huts were less than the proportions killed in the cement huts regardless of treatment. At the application rate of 1 g/m^2^ the pirimiphos methyl CS B and BM variants performed equally well, killing significantly more *An. gambiae* (75% and 76%) than did the 0.5 g/m^2^ rates of CS B (42%) or CS BM (52%) (p<0.001). The pirimiphos methyl EC formulation killed only a small proportion of *An. gambiae* (23%).

The trend in mortality for *Cx. quinquefasciatus* in the mud lined huts mirrored that of *An. gambiae*. A slightly higher mortality rate was observed with the 1 g/m^2^ pirimiphos methyl BM (51%) than with the B (46%) variant (P = 0.04). The 1 g/m^2^ application rates of these formulations killed more *Cx. quinquefasciatus* than the 0.5/g^2^ rate (P = 0.001). With the pirimiphos methyl EC formulation, only 15% of *Culex* was killed overall.

### Residual Efficacy Against Free Flying Mosquitoes

#### Cement huts

**Figure 1:**
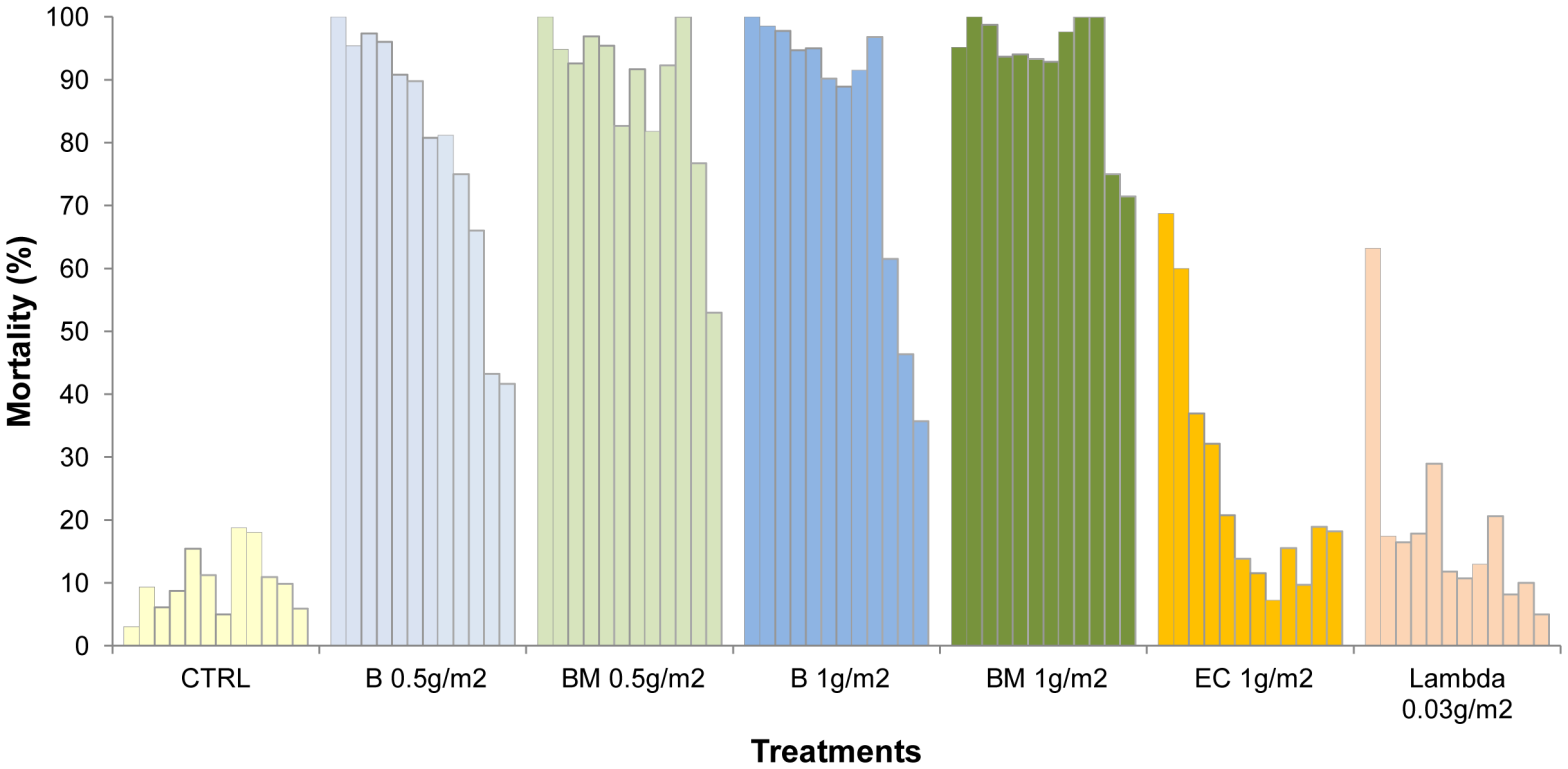
Monthly mortality rates of *Anopheles gambiae* in experimental huts lined with cement plastered walls.

**Figure 2:**
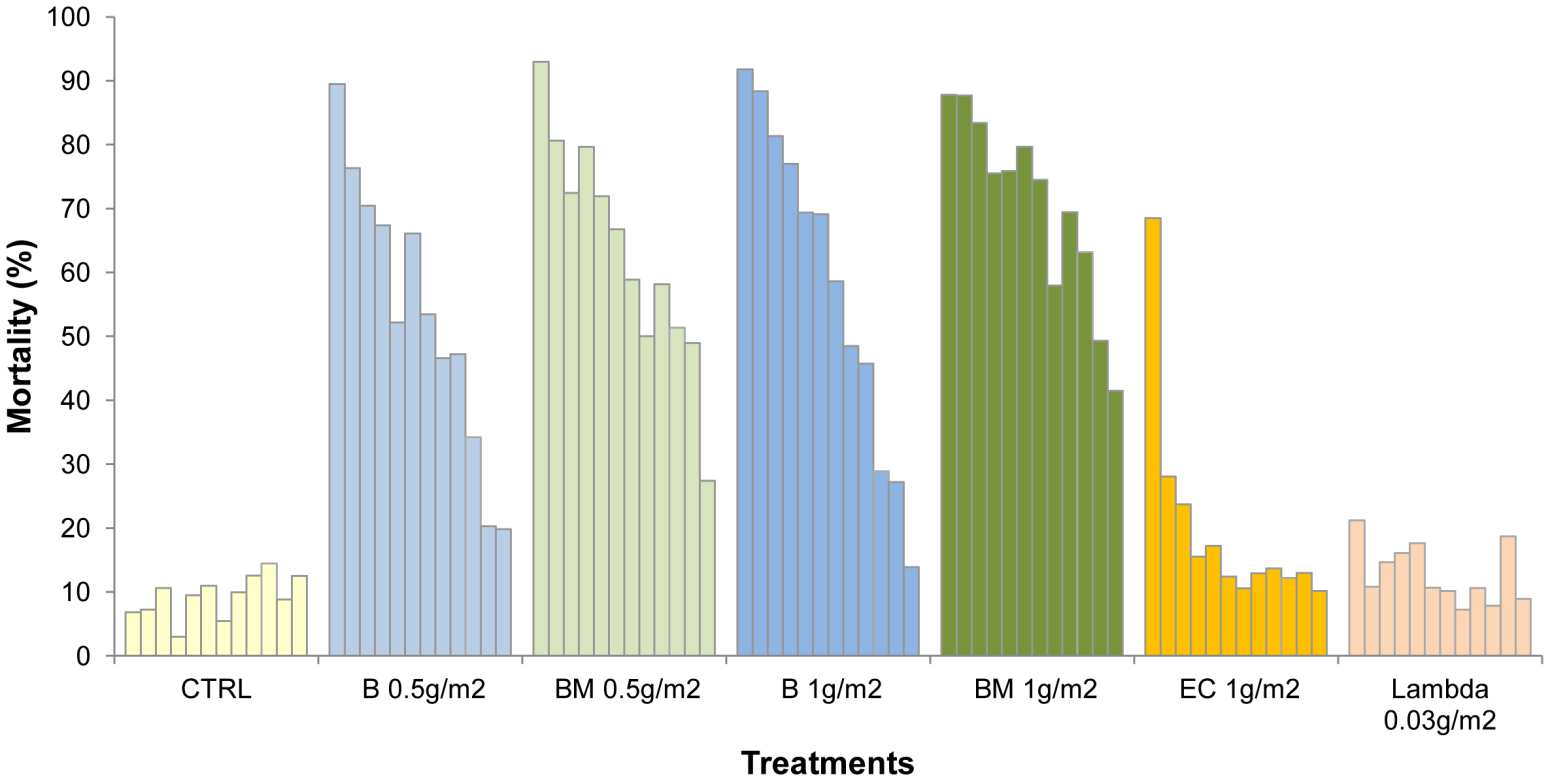
Monthly mortality rates of *Culex quinquefasciatus* in experimental huts lined with cement plastered walls.

Figures 1 and 2 show the monthly mortality rates of *An. gambiae* and *Cx. quinquefasciatus* that freely entered the cement walled huts during the course of the year.

The pirimiphos methyl CS BM formulation killed more than 80% of the *An. gambiae* per month for 9 months at the 1 g/m^2^ application rate and for 6 months at the 0.5 g/m^2^ rate, after which mortality progressively declined (P<0.001) (figure 1). With the pirimiphos methyl EC formulation percentage mortality was highest during the first two months (>60%) but then decreased suddenly. With the lambdacyhalothrin CS formulation the mortality rate decreased to less than 20% within a month.

The trend in mortality for *Cx. quinquefasciatus* in the cement huts was similar to *An. gambiae*, but the loss of activity against this species was faster than against *An. gambiae* (p = 0.001) (figure 2). Mortality rates with the pirimiphos-methyl CS treatments exceeded 80% during the first month and remained higher than 60% after 6 months. With the pirimiphos-methyl EC treatment, efficacy was lost within just a month. With the lambdacyhalothrin CS treatment, efficacy was less than 20% after one month.

#### Mud huts

**Figure 3:**
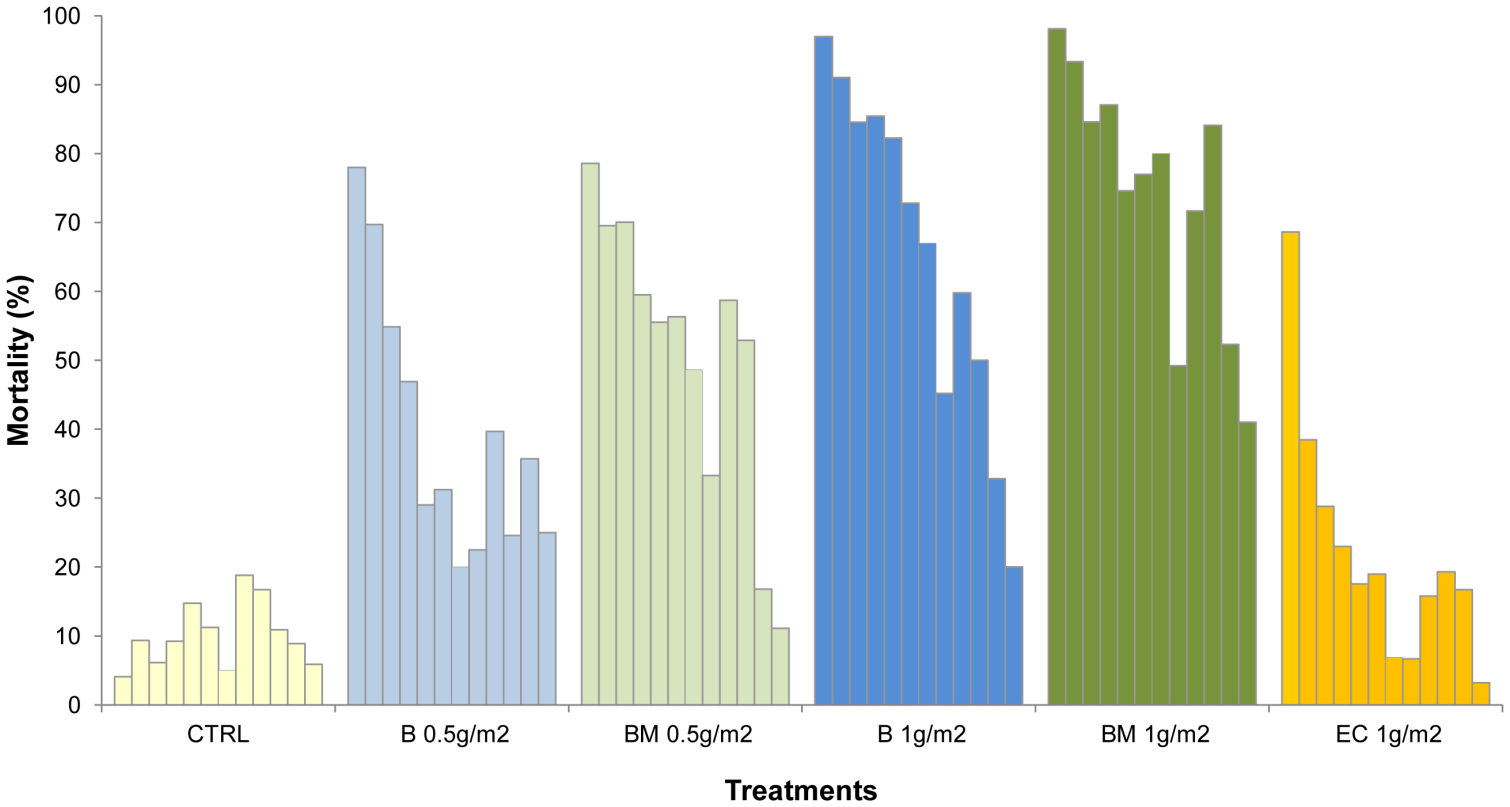
Monthly mortality rates of *Anopheles gambiae* in experimental huts lined with mud plastered walls.

**Figure 4:**
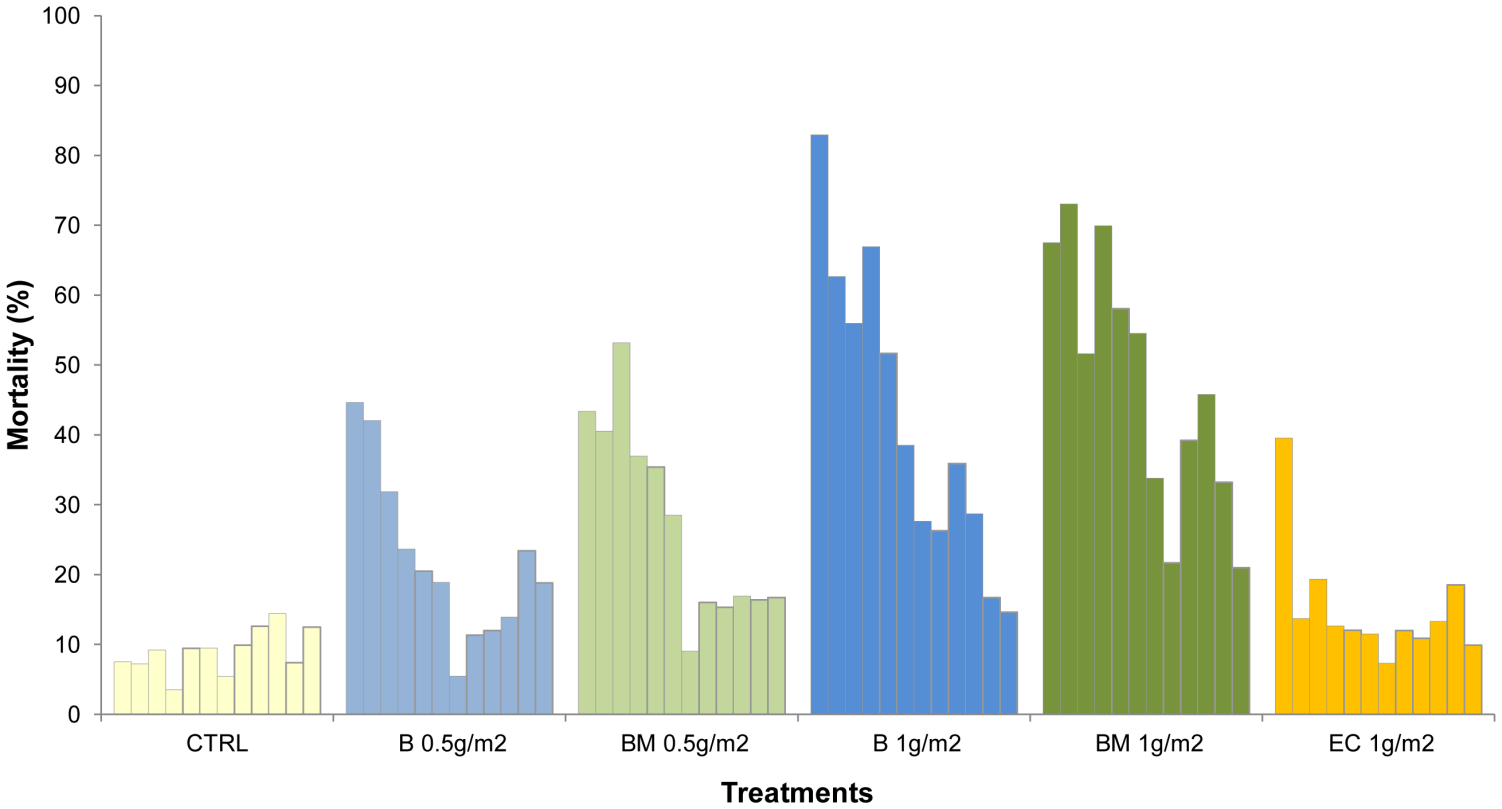
Monthly mortality rates of *Culex quinquefasciatus* in experimental huts lined with mud plastered walls.

Figures 3 and 4 show the monthly mortality rates of *An. gambiae* and *Cx. quinquefasciatus* in the mud lined huts over one year.

The rate of treatment decay was faster in the mud walled huts than in the cement walled huts (p<0.001). The 1 g/m^2^ application rates of the pirimiphos methyl CS B and BM variants killed more than 80% of *An. gambiae* per month for 4–5 months before showing a progressive decrease in activity. Apart from an unusual drop in mortality at month 8, which was observed with all formulations, the 1 g/m^2^ pirimiphos methyl CS BM produced more than 70% mortality per month for 10 months and the pirimiphos methyl B produced more than 60% mortality per month for 9 months. The difference in activity between the BM and B variants was more evident with the 0.5 g/m^2^ application rate (figure 3).

Only the 1.0 g/m^2^ application rate of pirimiphos methyl CS produced high mortality of *Cx. quinquefasciatus* for 6 months or longer (figure 4).

The pirimiphos methyl EC was only effective during the first month of application, killing nearly 70% of *An. gambiae* and 40% of *Cx. quinquefasciatus* before showing a sudden loss of activity.

The temporary drop in mortality around the 7–8^th^ month occurred at a time of year (November) when rainfall and humidity were low and the temperatures were rising.

### Species Characterization and Resistance Status

Only *An. gambiae* s.s. M form was found in the trial area. A discriminating concentration of 1% pirimiphos methyl (i.e. double the LC100) was established using a range of concentrations against the *An. gambiae* Kisumu strain (table 3). Using this discriminating concentration against *An. gambiae* adults collected as larvae from the trial site the mortality rate was 100% indicating no resistance. In WHO susceptibility tests with 0.05% deltamethrin test papers percentage mortality was 20% for *An gambiae* and 17% for *Cx quinquefasciatus* (table 4). In molecular assays on *An. gambiae* the frequency of *kdr* was 0.86 but no *Ace.1R* allele was detected. No molecular assays were conducted on contemporary samples of *Cx quinquefasciatus* but in an earlier characterization in the area the frequency of *kdr* was 0.63 and the frequency of *Ace.1R* was 0.03 [23].

**Table 3:** Determination of diagnostic concentration of pirimiphos methyl in silicon oil in WHO susceptibility tests. Mosquitoes were exposed for 1 h and mortality recorded 24 h later.

Strain	% Concentration	Number tested	% Mortality
*Anopheles gambiae* Akron (F1 of wild population)	0	71	0
	0.125	83	9
	0.25	94	58
	0.50	90	100
	1.0	92	100
*Anopheles gambiae* Kisumu (laboratory susceptible strain)	0	72	0
	0.125	89	18
	0.25	73	99
	0.50	93	100
	1.0	95	100

**Table 4:** Susceptibility of wild *Anopheles gambiae* and *Culex quinquefasciatus* from field station Akron to pyrethroids.

Species	Number tested	% Mortality
*Culex quinquefasciatus*	96	17
*Anopheles gambiae*	80	20

F1 adults were exposed to 0.05% deltamethrin test papers for 1 h and mortality recorded 24 h later.

### Residual Bioassay Activity

**Figure 5:**
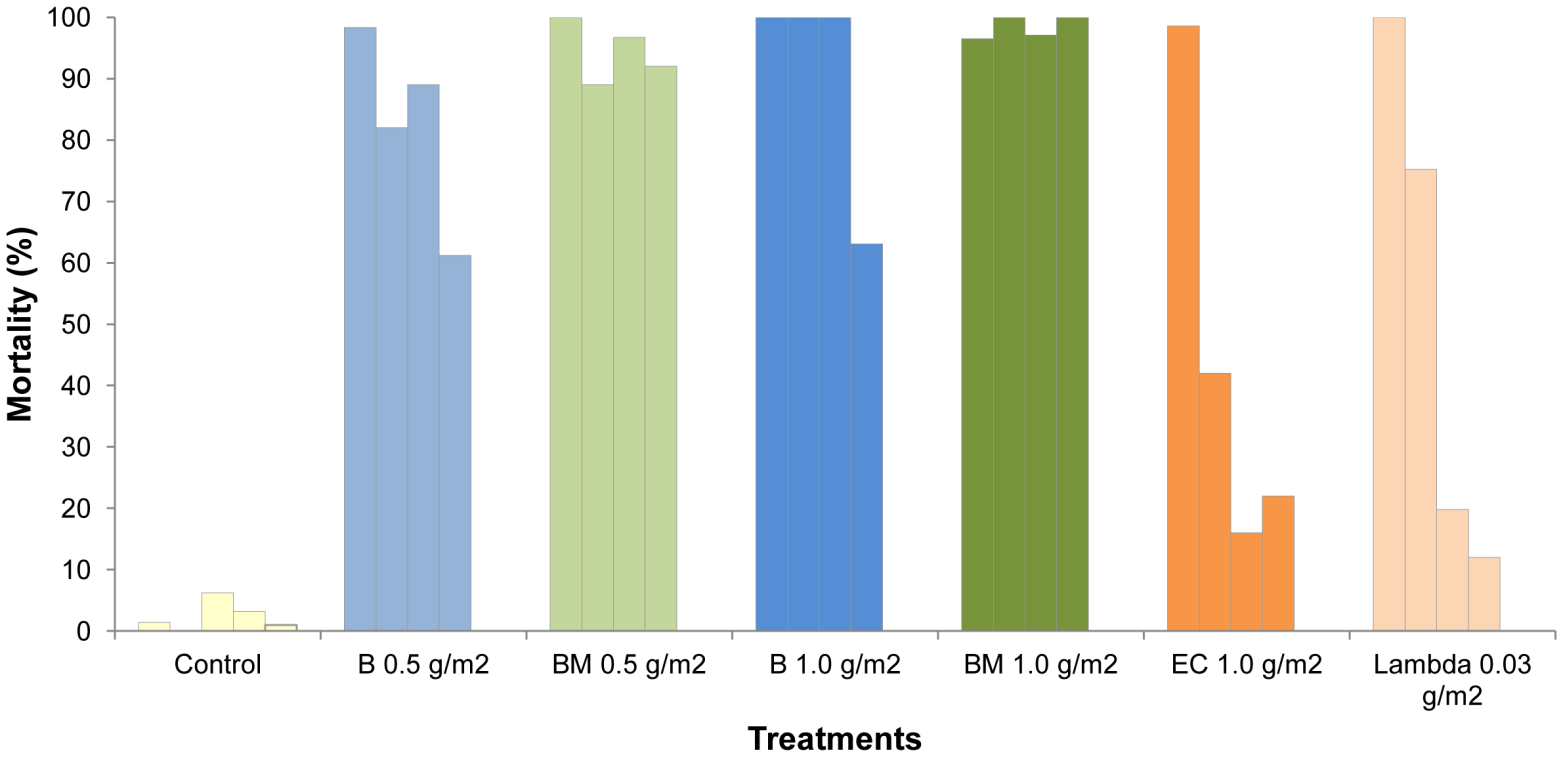
Residual activity of IRS in experimental huts lined with cement plastered walls using WHO cone bioassays and insecticide susceptible *Anopheles gambiae* Kisumu strain.

**Figure 6:**
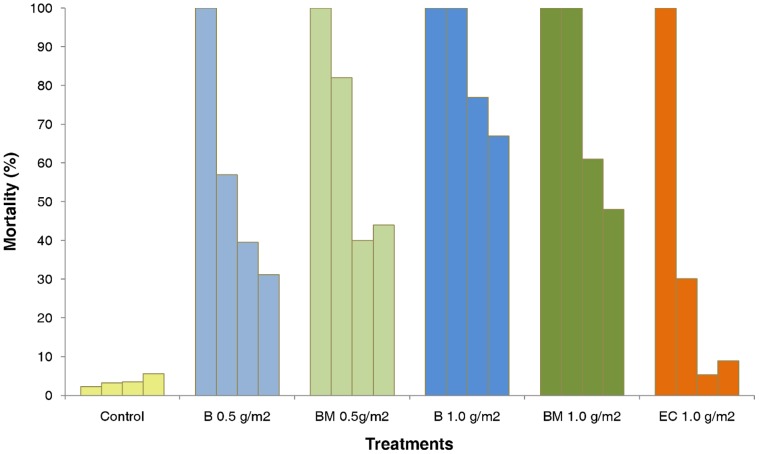
Residual activity of IRS in experimental huts lined with mud plastered walls using WHO cone bioassays and insecticide susceptible *Anopheles gambiae* Kisumu strain.

Figure 5 and 6 show the results of residual bioassay tests conducted with the susceptible *An. gambiae* Kisumu strain on insecticide treated cement and mud wall surfaces.

On cement surfaces the BM CS variant killed more than 80% throughout the 4 quarters. The B CS variant killed more than 80% during the first three quarters but by the fourth quarter mortality had decreased to 60% (figure 5). The EC formulation produced a faster decline in activity with mortality decreasing from 100% to 40% after the first quarter (P<0.001). The lambdacyhalothrin treatment was highly active on cement for 6 months, showing 75% mortality of susceptible *An. gambiae* during the second quarter; this contrasts with a low mortality rate over the same period against free flying wild *An. gambiae* in the lambdacyhalothrin hut.

The residual activity of all treatments on mud walls was high initially (100% mortality in the first quarter) but decreased steadily over time (figure 6). A decrease of B and BM activity was evident during the second quarter for the lower dosage and during the third quarter for the higher dosage (p<0.001). The EC was ineffective by the second quarter.

### Responses of Sleepers to the Treatments

All odour due to treatment began to fade within a day of spraying and after 6 days no odour was reported by sleepers for any treatment. Odour was dosage dependent and all volunteers agreed that pirimiphos methyl EC produced the strongest odour. Odour evoked a variety of responses from good to unpleasant but strong odour was not always deemed negatively but as indicating a good product.

## Discussion

With impetus from governments, the Global Fund and President’s Malaria Initiative, IRS is being deployed in 31 African countries in the attempt to reduce malaria burdens across Africa [1]. Of the four classes of insecticide currently available for IRS, the cheapest, DDT, is compromised by its negative environmental impact, and the most widely used, the pyrethroids, threaten to accelerate the selection of resistance and undermine long lasting insecticidal nets [21]–[22], [31]. The WHO is recommending switching from IRS with pyrethroid in any country where LLINs are widely used in order to sustain the operational life of the LLIN strategy [32]. There is an urgent need to find alternative insecticides to pyrethroids and DDT in order to maintain the two pronged attack of IRS and LLIN against malaria while simultaneously reducing the selection pressure for pyrethroid resistance. The President’s Malaria Initiative is using the carbamate bendiocarb for IRS in countries where pyrethroid resistance is prevalent [4]–[5], [33]–[35]. The problem with carbamate insecticides is their short residual life, which means having to spray twice per year in countries where malaria transmission seasons are longer than the 2–3 month residual lifespan of the carbamate, as occurs in many sub-Saharan countries [36]–[37]. Short residual life is a problem shared with the fourth group of insecticides used for IRS, the organophosphates. As shown in the present study, standard EC formulations of organophosphates will last for 2–3 months at best and this restricts their deployment by national malaria control programmes [2]. Microencapsulation of organophosphates minimizes that limitation. Residual activity is extended for up to 10 months on cement walled surfaces and for 6–8 months on mud walled surfaces, which is sufficient to cover the longest transmission season or even the twice yearly transmission seasons that occur in East Africa [31], [38]. While an application rate of 0.5 g/m^2^ might be sufficient in more modern housing which have cement walls, an application rate of 1.0 g/m^2^ would be necessary in rural villages where mud lined walls are standard.

The microencapsulation technology that enabled the development of lambdacyhalothrin CS, a long lasting pyrethroid formulation, provided the stimulus for Syngenta’s development and re-formulation of the organophosphate pirimiphos methyl. The evolution of resistance, as shown in the present hut trial with pyrethroid IRS, can undermine the residual life that encapsulation would otherwise bring to a compound. Lambdacyhalothrin CS has a residual life of at least 6 months on cement surfaces, as shown in bioassays with susceptible mosquitoes, but once wild mosquitoes become highly resistant to pyrethroids, the duration of protection may be reduced to a month or less, despite surface residues persisting for much longer.

In such conditions only an insecticide that shows no cross resistance to pyrethroids, such as an organophosphate or carbamate, can be deployed to good effect [4], [24], [37]. The longer the residual activity the better the cost-benefit. Recurrent malaria control campaigns over 3 years with the carbamate, bendiocarb, in Benin had a significant impact on transmission by the malaria vector *An gambiae* which was pyrethroid resistant [4], [34]. However, in a community randomized trial that compared the combined impact of bendiocarb IRS plus LLINs against LLINs alone, the bendiocarb IRS showed no additional impact over LLINs alone [39]. After an IRS campaign with bendiocarb on Bioko island in Equatorial Guinea, the prevalence of malaria started to increase 3–5 months after spraying as bendiocarb residues decayed [36]. Microencapsulated IRS products with longer residual activity than bendiocarb have the potential to control malaria over an entire transmission season while covering any break in control resulting from shorter lived insecticides.

Providing universal coverage of LLINs to populations at risk has become a priority for many national malaria control programmes in recent years [1]. Pyrethroid resistant Anophelines are now present in all African countries, and the frequency of resistance is increasing due to selection pressure from LLINs and pyrethroid IRS [12]–[17]. LLINs can still provide personal protection particularly when the netting is intact [40]. With the increasing coverage of LLINs in many endemic countries the deployment of IRS using pirimiphos methyl CS will usually be done against a background of LLIN coverage. Case control studies have shown that a combined intervention of organophosphate IRS and pyrethroid treated net provides added protection against malaria infection [41]. Recent trials of the combination intervention of LLINs and pirimiphos methyl CS in experimental huts against pyrethroid resistant *An gambiae* support that inference (Ngufor & Rowland, unpublished).

In some countries where extra resources for malaria control have become available, programmes are deploying both IRS and LLIN in areas of high transmission. There is some evidence that the combined approach can potentially provide greater protection or reduce transmission faster than one method alone [40], [42]. The combined use of organophosphate IRS and LLINs, through the deployment of two classes of insecticide with alternative modes of action has the potential to reduce selection pressure for resistance [32]. Pyrethroid resistant mosquitoes that come into contact with IRS surfaces should be killed by the organophosphate. The spatial combination of pirimiphos methyl CS and LLINs within homes may help to keep malaria control and elimination targets on track. Whether the combination intervention will delay the selection of resistance is less certain and will depend in part on the initial frequency of each type of resistance.

Limited environmental persistence and long residual activity makes pirimiphos methyl CS a more attractive prospect than DDT for IRS programmes [43]. There is a need to accelerate the development, registration and global access to pirimiphos methyl CS. Any hope or plan for sustained control or indeed elimination of malaria in sub Saharan Africa will depend on continued investment and development of long lasting insecticides for vector control.
